# Cervical Visual Inspection with Acetic Acid (VIA) and Oncogenic Human Papillomavirus Screening in Rural Indigenous Guatemalan Women: Time to Rethink VIA

**DOI:** 10.3390/ijerph182312406

**Published:** 2021-11-25

**Authors:** Anne Jeffries, Consuelo M. Beck-Sagué, Ariel Bernardo Marroquin-Garcia, Michael Dean, Virginia McCoy, Diego Aurelio Cordova-Toma, Eric Fenkl, Purnima Madhivanan

**Affiliations:** 1 Robert Stempel (RS) College of Public Health and Social Work, Florida International University (FIU), Miami, FL 33199, USA; anneffries@gmail.com (A.J.); mccoyh@fiu.edu (V.M.); eafenkl@gmail.com (E.F.); 2 Partner for Surgery, McLean, VA 22101, USA; ariel@companerogt.org (A.B.M.-G.); diegoaureliocordova@gmail.com (D.A.C.-T.); 3 Laboratory of Translational Genomics, National Cancer Institute (NCI), National Institutes of Health, Rockville, MD 20850, USA; deanm@mail.nih.gov; 4 Mel and Enid Zuckerman College of Public Health, University of Arizona, Tucson, AZ 85724, USA; pmadhivanan@arizona.edu

**Keywords:** cervical cancer screening, visual inspection with acetic acid, human papillomavirus, cytology, cryotherapy, low-resource settings, low- and middle-income countries, Guatemala

## Abstract

Single-visit “screen-and-treat” strategies using visual inspection with acetic acid (VIA) and cryotherapy (liquid nitrous oxide ablation) in low-resource settings are commonly used to detect and treat precancerous lesions for cervical cancer prevention. This study compared VIA sensitivity and specificity in rural indigenous Guatemalan communities, to that of oncogenic human papillomavirus (HPV) testing for detection of precancerous changes, using cytology as the reference standard. Between 3–8 September 2017, trained nurses examined 222 women aged 23–58 years with VIA. Specimens for liquid-based cytology and HPV testing were obtained prior to VIA with a cytobrush and transported in PreservCyt to a US clinical laboratory. VIA and HPV test sensitivities were assessed as proportions of women with abnormal cytology that had abnormal VIA or HPV results, respectively, and specificities, as proportions with normal cytology with normal VIA or negative HPV tests. Of 222 women, 18 (8.1%) had abnormal cytology (1 carcinoma in a participant who received VIA-based cryotherapy in 2015, 4 high- and 5 low-grade squamous intraepithelial lesions, and 8 atypical squamous cells of undetermined significance (ASCUS)). Excluding ASCUS, sensitivities of VIA and HPV were 20.0% and 100%, respectively. VIA-based screening may not be acceptable for detecting precancerous lesions, and field cryotherapy for preventing malignancy. The World Health Organization recommended in 2021 “…using HPV DNA detection as the primary screening test rather than VIA or cytology”.

## 1. Introduction

Cervical cancer incidence and mortality declined in high-income countries with the widespread use of cytological screening and treatment of precancerous lesions [1]. Timely detection and treatment can prevent progression of precancerous lesions to cancer, preserving life and fertility. Oncogenic human papillomavirus (HPV) types are present in 95–100% of cervical cancer specimens and cause most cases of cervical cancer [2]. The Latin America and Caribbean (LAC) region is home to just 9% of the world population but bears approximately 16% of the world cervical cancer mortality [3,4]. Low access to screening and treatment, particularly in remote rural populations, are important barriers to timely detection and treatment. Cervical cancer mortality in Guatemala is the leading cause of cancer mortality among women aged 15–44 years and is reportedly rising in most age groups [5], in contrast to the cervical cancer mortality in other LAC countries.

In low- and middle-income countries lacking access to standard-of-care cervical cytology nationwide, visual inspection with acetic acid (VIA), with onsite cryotherapy (liquid nitrous oxide ablation to freeze and destroy precancerous tissue at −50 degrees Celsius), (“test-and-treat”), have been recommended [6,7]. VIA requires minimal equipment; acetic acid (vinegar) and a speculum to perform the exam. VIA does not always allow examiners to accurately “grade” lesions, but examiners can treat visible acetowhite lesions with cryotherapy, thus reducing the need for multiple visits [7]. Recently, assumptions about the impossibility of integrating complex health interventions in low- and middle-income country health programs have been reexamined, based on the success of the antiretroviral therapy scale-up for the management of HIV [8]. LAC healthcare professionals are rethinking the appropriateness of low-technology strategies and of adapting and adopting more technically advanced interventions for improved detection and treatment. This analysis compared the performance of VIA by trained nurses to HPV testing, relative to liquid-based thin-layer preparation cytology as the reference standard in rural indigenous communities in Guatemala.

## 2. Materials and Methods

### 2.1. Study Participants

Women living in underserved rural indigenous communities in Alta and Baja Verapaz, Guatemala with little access to clinical facilities are advised by community health workers 14–21 days prior to community-based screening activities that VIA will be offered at no cost by Partner for Surgery, a non-governmental organization, which provides multiple health services in rural villages, including cervical cancer screening and precancerous lesion cryotherapy treatment to women aged more than 21 years. VIA screening in these and other rural villages in Guatemala, with recruitment conducted in this way, is routinely offered by Partner for Surgery several times per year. VIA examinations take place in community centers in the villages, which allows privacy for the women to be interviewed (most through multilingual Spanish/Mayan language interpreters) and examined by Guatemalan registered nurses trained and experienced in VIA and liquid preparation collection.

A sterile, disposable clear plastic vaginal speculum is inserted in the vagina and freshly prepared 4% acetic acid is applied to the cervix for cytology. After one minute, the cervix is inspected using a light source (a hand-held flashlight). VIA examinations are considered positive for precancerous changes when a well-defined, dense whitened area with regular margins is visible at the squamocolumnar junction or in the transformation zone. VIA tests are considered negative if no acetowhitening is observed. Cryotherapy is offered to women with abnormal VIA exams. Those whose VIA examination suggests malignancy (e.g., acetowhite lesion is raised and irregular, or bleeding on contact) are referred on the day of the examination to specialized care in a provincial secondary care or capital (Guatemala City) tertiary facility offering subsidized but not free care.

### 2.2. Study Procedures

VIA examination procedures took place in the villages visited from 3–8 September 2017 (Rabinal, Tactic, Compur, and Cahabon, in Alta and Baja Verapaz, Guatemala), with invitations 14–21 days before, in an identical way as is routine (described above), except that specimen collection was performed immediately after speculum insertion, before application of acetic acid. Specimen collection for cytology and HPV testing was completed prior to VIA using spatulas and cyto-brushes. Specimens were placed immediately after being obtained into PreservCyt, a methanol-based transport solution. Containers were labeled with a number to link with the patient identifiers, VIA exam, and other information. Specimens were transported to the Palm Beach Pathology Laboratory (West Palm Beach, FL, USA), a US Clinical Laboratory Improvement Amendments (CLIA) approved laboratory, where all laboratory testing was performed. The cytological specimens were processed using Thin Prep Processor 2000 (Hologic Corp., Marlborough, MA, USA) liquid-based thin-layer preparation, and results were reported using Bethesda System for Reporting Cervical Cytology. HPV testing (which identified HPV 16 and 18, grouping all other oncogenic HPV types as “high risk”) was performed by nucleic acid amplification testing using Aptima HPV 16 18/45 Genotype Assay (Hologic Corp., USA). Results of cytological tests and HPV tests were provided to Partner for Surgery staff within 45 days of specimen collection.

Partner for Surgery advised patients to seek specialized care in government facilities if they had abnormal results (e.g., high- and low-grade squamous intraepithelial lesions (HSIL, LSIL), positive HPV tests, etc.). The referral was to include VIA results and other results provided to Partner for Surgery staff to distribute to patients. After linkage to cytological and HPV test data, and the distribution to Partner for Surgery staff and to participants as described above, all identifiers were removed from the VIA, cytology, HPV test, and interview data, and the data were provided for analysis to authors.

### 2.3. Statistical Analysis Methods

This was a cross-sectional analysis of the de-identified data collected during the brief interviews of women who presented for VIA (including age, pregnancy and births, and past VIA examination and treatment history), and results of VIA, HPV testing, and cytological examination. Abnormal cytology results were defined as those that reported HSIL, LSIL, carcinoma, or atypical squamous cells of unknown significance (ASCUS). Due to cytological and HPV testing being more likely to signify persistent or progressive HPV infection (vs. transient infection that would clear spontaneously) in immunocompetent older women than in younger women [9,10], we compared the prevalence of abnormal VIA, cytological, and HPV tests by age-group (less than 30 years vs. 30 years and older). Prevalence was calculated by dividing the number with an abnormal or positive result for each age group by the total number in each group. To assess the strength of association between younger age and abnormal results, prevalence ratios were calculated by dividing the prevalence in women aged less than 30 years over the prevalence in women aged 30 years and older. Precision of prevalence ratio estimates and statistical significance were assessed using 95% confidence intervals (95% CIs); statistical significance was also assessed using chi square tests (or, if at least 1 expected cell was less than 5, by Fisher exact two-tailed tests). All analyses were performed with Epi Info v. 3.5.4 (Centers for Disease Control and Prevention, Atlanta, GA, USA) and OpenEpi.com [11,12].

VIA and HPV test sensitivity and specificity were assessed for all participants and restricted to only women aged 30 years and older using cytological exam results as the reference standard. Analyses were performed excluding and including ASCUS results. VIA and HPV test sensitivities were calculated as the proportions of women with abnormal cytology specimens whose VIA test was abnormal, or whose HPV test was positive, respectively [13]. Specificities for VIA and HPV testing were calculated as the proportions of these tests that were normal (VIA) or negative (HPV tests) among women with normal cytology specimens; 95% CIs were used to assess precision of estimates. Predictive values of positive VIAs and HPV tests were calculated by dividing the number of patients with an abnormal VIA or positive HPV test who also had abnormal cytologies by the total number with an abnormal VIA or positive HPV test, respectively. Predictive values of negatives were calculated by dividing the number of patients with normal VIA or negative HPV who also had normal cytologies by the total number of patients with normal VIAs or negative HPVs, respectively. Diagnostic accuracy was defined as proportion of VIA or HPV tests in which the diagnosis was correct.

### 2.4. Sample Size Estimation

Data used for estimation of sample size consisted of results of de-identified HPV tests collected during VIA activities in Guatemala indigenous communities, as part of the National Cancer Institute (NCI) international HPV infection prevalence surveys in 2013. In that dataset, 29.6% had HPV detected in their samples. For 80% power to detect a statistically significant difference between a minimum HPV test sensitivity of 95% and a maximum VIA sensitivity of 60%, with two-sided significance level of 95%, we would require 22 women with abnormal cytological examinations (HSIL, LSIL, or carcinoma) [11,12]. Anticipating approximately 10% of participants to have an abnormal cytological examination, and over 20% to have HPV detected, we sought to obtain specimens from at least 222 women who were examined by VIA.

## 3. Results

Nurses examined 223 women aged 23–58 (median age = 36; interquartile range = 30–43) years. Parity ranged from 1–12 deliveries, and 96% had delivered at least 1 child; 11 (4.9%; 95% CI = 2.5–8.7%) of VIA examinations were considered abnormal, including 1 with a lesion consistent with cancer, in a woman who had had VIA and cryotherapy in 2015. Oncogenic HPV types were detected in 38 (17.0%; 95% CI = 12.3–22.6%) women. Of the 222 women with usable cytological specimens, 10 (4.7%; 95% CI = 2.3–8.5%) had abnormalities, including the carcinoma, 5 LGSIL, and 4 HGSIL. Another 8 had ASCUS. HPV prevalence was significantly higher among women aged less than 30 years than among older women (Table 1). Younger women were more likely to have an abnormal cytological examination (not statistically significant) or abnormal VIA (approached but did not achieve statistical significance (Table 1).

HPV testing detected all 10 (sensitivity = 100%) participants with HGSIL, LGSIL, and the 1 with cancer, while VIA tests detected only 2 of 10 (sensitivity = 20%; *p* = 0.007) (1 LGSIL and the 1 with cancer) (Table 2a). Negative predictive values for HPV testing and VIA in were 100% and 96.1%, respectively. Both women with cytology abnormalities detected by VIA (one with carcinoma, another with LGSIL) were referred for specialized care on the day of the screening activity, the latter after declining cryotherapy. Specificities for HPV testing and VIA were 181/204 (88.7%) vs. 196/204 (96.1%; *p* = 0.005), respectively. VIA did not detect any participants with ASCUS, but HPV testing detected five of eight; sensitivities of HPV and VIA for detecting abnormal cytologies, including ASCUS, were 83.3% and 11.1%, respectively (*p* < 0.0001). When confined to participants aged ≥30 years, HPV test sensitivity (excluding ASCUS) remained 100%, and the 95% CI still did not overlap with VIA sensitivity, which declined to 16.7%; both specificities rose (to 90.5% for HPV and to 97.4% for VIA), still significantly different (95% CIs did not overlap) (Table 2b). Diagnostic accuracy of HPV testing did not differ significantly from that of VIA.

Cryotherapy was used to treat four women with abnormal VIA exams who did not have abnormal cytology or HPV detected. Another woman was referred based on abnormal VIA and had a negative HPV test as well as an unsatisfactory cytological specimen and was not included in this analysis.

## 4. Discussion

In this small study, sensitivities of VIA and HPV testing differed considerably relative to cytology, more than anticipated. Only 2 of 10 women with abnormal cytological examinations had abnormal VIA; 1 of these 2 had had an abnormal VIA and cryotherapy 2 years before and had progressed to invasive cancer despite the cryotherapy. Even though only 10 participants had cytology examinations meeting the criteria for carcinoma, HGSIL or LGSIL, the difference in sensitivity between VIA and HPV achieved statistical significance (Table 2), due to the low sensitivity of VIA, lower than expected. These findings are concerning; they suggested that the sensitivity of VIA for cervical cancer screening was unexpectedly low, and that VIA-guided cryotherapy did not appear to reliably affect the course of a precancerous lesion. Inclusion of women aged less than 30 years increased the prevalence of abnormal results in HPV PCR and VIA, as well as in cytology, possibly by the detection of infections that may have been spontaneously cleared with time [9,10]. This association achieved statistical significance only in the HPV testing, due to the low numbers of women with abnormal VIA and cytology, hence the low power of our study for the age-group analysis. As expected, confining the analysis only to women aged more than 30 years resulted in modest increases in specificity for HPV (from 88.7% to 90.5%) but in further decline in sensitivity (from 20.0% to 16.7%) for VIA.

These findings suggested that alternatives to VIA should be sought, and that HPV testing should be considered. The assessment that opens a 2008 review of new approaches to cervical screening LAC was grim: “Efforts to control cervical cancer in the LAC have been largely unsuccessful.” [14]. The ageing population of LAC, and the decline in other causes of death for women, particularly pregnancy-related mortality, probably have contributed to the current trends in cervical cancer mortality, particularly in Guatemala [5]. VIA has been increasingly recognized as having serious problems with sensitivity, and cryotherapy, with efficacy [15,16]. The encouraging results of VIA-based and HPV-based screening equivalency from well-executed long-term randomized trials following patients in the past [17] have been difficult to reproduce in real-world settings where the superiority of HPV-based screening and its feasibility in low- and middle-income settings has been documented for over 10 years [15].

In considering HPV-based screening as an alternative to VIA, concerns arise related to the possible loss of cost-effectiveness with HPV screening, and the loss of the “screen-and-treat” efficiency of VIA, in the context of resource-constrained settings due to the potential requirement to send specimens to a laboratory and requirement for a return visit for treatment. However, the ASPIRE trial, which assessed the feasibility and acceptance of HPV self-collection vs. VIA in a cohort of women from Kisenyi, Uganda, explored cost-effectiveness [18], reporting that using self-obtained samples for HPV nucleic acid amplification testing within an HPV screen-and-treat program was the most cost-effective strategy, adding that “even if the cost of the test were increased, short of quadrupling these costs”, in this population, it would remain the most cost-effective strategy [18]. The concern about the delay implicit in sending specimens to a laboratory, processing, and returning with results was addressed in a Cameroon study, where real-time PCR identified women for biopsy and endocervical curettage and was found suitable for one-contact “test-and-treat” strategies [19] Cost and test performance data from the START-UP demonstration projects in India, Nicaragua, and Uganda were used to evaluate the cost-effectiveness of various screening strategies; the use of the *care*HPV test (QIAGEN, Gaithersburg, MD, USA), a lower-cost DNA test to be used in settings without clean water or electricity, was the most cost-effective strategy [20].

This study had several limitations. Abnormal cytology and VIA examination prevalence in the population were lower than expected; the low number of positive cytology and VIA examinations reduced the power to detect significant differences by age-group (Table 2a) and the precision of sensitivity estimates (Table 2b). Furthermore, the use of cytological testing as the reference standard may underestimate HPV test specificity. Most studies of cervical cancer screening use biopsy evidence of abnormalities as the reference standard, a limitation of our study. However, obtaining biopsies of participants was obviously not feasible in a community-based screening project in indigenous villages. Moreover, cytological examinations remain the most used screening method worldwide, including high-income countries, and have been credited with much of the reduction in cervical cancer mortality worldwide [1]. As such, in the absence of the biopsy evidence, cytological examination results were considered an appropriate option. Another concern was that many women traveled 3–4 h by foot to attend. Some older or ill women may have been unable to attend despite wanting to do so, potentially reducing the proportion of older women who were 30 years old and older and possibly exaggerating the prevalence of persistent or progressive high-risk HPV infection.

Since sexual debut may be early in these communities, as the high parity suggests, and cervical cancer is the principal cause of cancer mortality in Guatemala in 15–44 year old women, Partner for Surgery routinely offers VIA screening to women aged over 21 years. Although the prevalence of high-risk HPV infection in younger participants suggested that some of these infections may have regressed spontaneously, some of the participants with high-grade dysplasia were less than 30 years old, supporting the Partner for Surgery approach. Moreover, even the analysis confined to women aged 30 years and older, although it excluded some participants with high-grade dysplasia, showed significantly higher HPV sensitivity than VIA. Finally, overall test accuracy, which tends to overstate lower-sensitivity test performances in low prevalence populations [13], did not differ significantly between HPV and VIA.

Despite these limitations, our study suggested that VIA-based screening with cryotherapy may miss women with treatable precancerous lesions. The failure of cervical cancer mortality to decline (and indeed, the apparent increase in mortality) in Guatemala in the decades in which VIA has been widely used [5] suggested the need to explore alternatives, including point-of-care HPV-based screening, which in rural India decreased advanced cervical cancer and its related deaths by over 40%, without significant reductions seen using either cytological or VIA-based screening in a head-to-head trial [15]. Use of self-obtained samples for HPV PCR is particularly promising in the Guatemalan rural indigenous population [21]. Other emerging alternatives to VIA-based screening include mobile colposcopy for examination of HPV- or VIA-positive patients [22], or for primary screening with real-time interpretation using a machine learning-based interpretation where the enhanced visual assessment image [23,24] is to be integrated in a point-of-care protocol, although this latter option does not have any well-documented successful field evaluation. HPV testing, with or without mobile colposcopy, which can be used even without real-time machine learning-based interpretation, should be considered for adoption for cervical cancer screening and control in underserved rural indigenous communities in Guatemala.

## 5. Conclusions

This analysis of anonymized data comparing VIA “test-and-treat” screening to HPV testing using cytological examination as a reference standard suggested that VIA performed by well-trained and highly skilled staff may be unreliable because of lower sensitivity. Furthermore, failure of cryotherapy to arrest the progress of pre-cancerous lesions in one of the indigenous women living in rural underserved communities in Guatemala suggested that cryotherapy in the field may need to be reconsidered as well. HPV testing, which can be performed in real time in the field on self-obtained samples [21], is being deployed in other low-resource settings worldwide. This small study suggested that there are opportunities to examine alternatives to relatively low-technology techniques that may address the persistent and growing problem of cervical cancer mortality in Guatemala. The World Health Organization recommended in 2021 “using HPV DNA detection as the primary screening test rather than VIA or cytology in screening and treatment approaches…” adding that “existing programs using VIA as the primary screening test should transition rapidly because of the inherent challenges with quality assurance” [25].

## Figures and Tables

**Table 1:** Differences in the prevalence of positive screening tests by participant age-group (less than 30 years vs. 30 years of age or older) at the time of visual inspection with acetic acid (VIA) in women participating in community-based cervical cancer screening, 2017, Alta and Baja Verapaz, Guatemala.

Number (%) with Characteristic		Total	Prevalence Ratio (95% CI) *	*p*-Value
Oncogenic human papillomavirus detected (%)				
Age <30 years	14 (26.4)	53	1.86	0.039 ^†^
Age >30 years	24 (14.2)	169	(1.04–3.3)	
Abnormal cytological examination (%)				
Age <30 years	6 (11.3)	53	1.59	0.330 ^‡^
Age >30 years	12 (7.1)	169	(0.63–4.04)	
Abnormal visual inspection with acetic acid (VIA) (%)				
Age <30 years	5 (9.6)	53	3.19	0.047 ^‡^
Age >30 years	5 (3.0)	169	(0.96–10.6)	

* 95% confidence interval. ^†^ Uncorrected Chi square. ^‡^ At least one expected cell was less than 5; Fisher exact test two-tailed *p*-values used.

**Table 2:** Screening test (oncogenic human papillomavirus (HPV) testing and visual inspection with acetic acid (VIA)) performance relative to reference standard (cytological exam) in women participating in community-based cervical cancer screening, 2017, including: (**a**) all participants and (**b**) participants aged 30 years and older.

**(a) All participants**		
**HPV Test**	**Estimate**	95% CI *
Sensitivity	100%	72.3–100%
Specificity	88.7%	83.7–92.4%
Positive Predictive Value	30.3%	17.4–47.3%
Negative Predictive Value	100%	97.9–100%
Accuracy	89.3%	87.6–95.1%
**VIA**	**Estimate**	95% CI *
Sensitivity	20.0%	5.7–51.0%
Specificity	96.1%	92.5–98.0%
Positive Predictive Value	20.0%	5.7–51.0%
Negative Predictive Value	96.1%	92.5–98.0%
Accuracy	92.5%	88.2–95.4%
**(b) Analysis restricted to participants aged 30 years and older**		
**HPV Test**	**Estimate**	95% CI *
Sensitivity	100%	61.0–100%
Specificity	90.5%	84.8–94.1%
Positive Predictive Value	28.6%	13.8–50.0%
Negative Predictive Value	100%	97.4–100%
Accuracy	90.8%	85.4–94.5%
**VIA**	**Estimate**	95% CI *
Sensitivity	16.7%	3.0–56.4%
Specificity	97.5%	93.6–99.0%
Positive Predictive Value	20.0%	3.6–62.5%
Negative Predictive Value	95.0%	90.5–97.5%
Accuracy	92.8%	87.9–95.8%

* 95% confidence interval.

## Data Availability

De-identified data analyzed in this study are available from the authors to qualified researchers on request as an Epi Info v 3.5.4 MDB file or as an Excel file.
